# Barriers and opportunities in assessing calls to emergency medical communication centre - a qualitative study

**DOI:** 10.1186/s13049-014-0061-3

**Published:** 2014-11-11

**Authors:** Veronica Lindström, Kristiina Heikkilä, Katarina Bohm, Maaret Castrèn, Ann-Charlotte Falk

**Affiliations:** Karolinska Institutet, Department of Clinical Science and Education Södersjukhuset and Academic EMS, Stockholm, Sweden; Karolinska Institutet, Department of Neurobiology, Care Sciences and Society, Stockholm, Sweden; Department of Health and Care Sciences, Faculty of Health and Life Sciences, Linneaus University, Kalmar, Sweden; Karolinska Institutet, Department of Clinical Science and Education and Section of Emergency Medicine Södersjukhuset, Stockholm, Sweden

**Keywords:** Emergency medical services, Emergency medical communication centre, Dispatch centre, Emergency medical dispatcher, Registered nurse, Assessment

## Abstract

**Introduction:**

Previous studies have described the difficulties and the complexity of assessing an emergency call, and assessment protocols intended to support the emergency medical dispatcher’s (EMD) assessment have been developed and evaluated in recent years. At present, the EMD identifies about 50-70 % of patients suffering from cardiac arrest, acute myocardial infarction or stroke. The previous research has primarily been focused on specific conditions, and it is still unclear whether there are any overall factors that may influence the assessment of the call to the emergency medical communication centre (EMCC).

**Aim:**

The aim of the study was to identify overall factors influencing the registered nurses’ (RNs) assessment of calls to the EMCC.

**Method:**

A qualitative study design was used; a purposeful selection of calls to the EMCC was analysed by content analysis.

**Results:**

One hundred calls to the EMCC were analysed. Barriers and opportunities related to the RN or the caller were identified as the main factors influencing the RN’s assessment of calls to the EMCC. The opportunities appeared in the callers’ symptom description and the communication strategies used by the RN. The barriers appeared in callers’ descriptions of unclear symptoms, paradoxes and the RN’s lack of communication strategies during the call.

**Conclusion:**

Barriers in assessing the call to the EMCC were associated with contradictory information, the absence of a primary problem, or the structure of the call. Opportunities were associated with a clear symptom description that was also repeated, and the RN’s use of different communication strategies such as closed loop communication.

## Background

The emergency medical communication center (EMCC) is usually the acutely ill patient’s first contact with the emergency medical services (EMS), and the EMCC can be described as the first link in the chain of survival for victims in out-of-hospital medical emergencies [1]. There is no doubt that the emergency medical dispatchers (EMD) in the EMCC play a crucial role in identifying critical medical conditions and in giving important instructions to the caller if the patient is suffering, for example, from cardiac arrest [2]. Early identification of the patient’s symptoms result in a better outcome for out- of-hospital cardiac arrest patients [3], and theoretically early identification may be important in other medical emergencies such as myocardial infarction [4] or stroke [5]. Previous studies have described the complexity of assessing an emergency call [3,6,7]. There are also studies identifying possible reasons for not identifying the caller’s need of care; language barriers [8-10] unnecessary questions asked by the EMD during the call [11], and lack of information from the caller [7]. Due to some of these difficulties, assessment protocols aiming to support the EMD’s assessment have been developed [12-14]. Despite continuous development of assessment protocols, studies show that the EMD identifies about 50-70% of patients who are suffering from cardiac arrest, myocardial infarction or stroke [2,4,5]. However, most previous studies have focused on the identification of specific conditions such as cardiac arrest and stroke, so it is still unclear whether there are any overall factors that may influence the assessment of the call to the EMCC. Therefore the aim of the study was to identify overall factors influencing the registered nurse’s assessment of calls to the emergency medical communication centre.

## Methods

### Study design

To achieve the aim of the study a qualitative study design was used and 100 purposeful selected calls to the EMCC were analysed using content analysis. A qualitative study design is suitable for studying interpersonal interactions; in this case, the interaction between a care-seeker who is calling the EMCC, and the RN assessing the call. The study was approved by the medical research ethics committee, Stockholm, Sweden in June 2008 (Dnr: 2008/810-31/2).

### Study context

This study was conducted in the county of Stockholm, which has a population of about two million. The regional County Council is responsible for the emergency medical services, and one single EMCC in Stockholm receives all medical, police and fire emergency calls. The EMCC also receives calls for the elective ambulance transport. The response to an emergency call to the emergency number 112 starts with a call-taker assessment that includes ascertaining the type of emergency and obtaining the caller’s address. Calls indicating medical problems are then immediately directed to a registered nurse (RN) for further assessment. The number of medical calls to the EMCC is approximately 315 000 per year, and out of these, around 150 000 calls result in dispatching an ambulance [15]. For calls for elective ambulance transport, the caller uses a separate phone number and is directly connected to an RN at the EMCC. The phone number for elective ambulance transport is used by patients, relatives and health care providers and the phone number is available to all the citizens in Stockholm [16]. The personnel assessing all types of medical calls to the EMCC are RNs who have additional education and training in: dispatching, interview techniques such as non-visual communication strategies, stress management, the computer system used at the EMCC, resource sharing, and how to give pre-arrival instructions such as telephone CPR. Annually, the RN’s at the EMCC receives at least 16 hours of continuing education and their skills and knowledge are re-tested every year. Whatever the type of medical call, the RN answers it. The RN assignment is to communicate with the caller (interview), assess the caller’s (patient’s) primary problem, decide the level of priority, and respond to the dispatcher who dispatches the resources [15]. To support the assessment of the call, an assessment protocol, the Swedish Medical Index, has been used since 1997. This assessment protocol is criteria-based and consists of 34 main chapters. It provides the RN with a series of questions to ask the caller [13]. Depending on the answers, the RN is advised, based on a fixed decision algorithm, how to triage the call and decide on the response. There are four levels of priority in the assessment protocol: Priority 1) acute life-threatening situation/condition; priority 2) acute but not life-threatening; priority 3) transportation to hospital; and priority 4) no medical need during transport [13]. If the call is assessed by the RN as a life-threatening situation/condition, a dispatcher is also connected to the call. The dispatchers’ task is to dispatch and direct the rescue unit. In the meantime, the RN who first assessed the call obtains the caller’s address and when possible gives instructions to the caller according to the type of emergency, and prepares him for the arrival of the ambulance. The dispatcher communicates with the ambulance personnel and provides them with relevant information regarding the assignment [15]. When the ambulance personnel arrive at the scene they send feedback via a handheld computer to the EMCC concerning their primary assessment of the patient’s problem and the priority level [17].

### Data collection

Purposeful case sampling was used for collecting data [18]. To archive a variety of data, two equal-sized groups of calls were collected, namely: 1) the first 50 calls to the EMCC dispatched as priority 1 where the ambulance nurse at the scene agreed with the priority and the assessment given by the RN at EMCC. An example; the ambulance assignment dispatched as patient with chest pain and highest priority (blue lights and sirens), and at the scene the patient’s primary problem assessed by the prehospital emergency care nurse was chest pain and the patient needed care with the highest priority. 2) 50 calls where the RN at the EMCC and the prehospital emergency care nurse at the scene gave a different assessment concerning the patient’s problem and the priority (under-triage). An example; the ambulance assignment dispatched as patient with unclear problems, lowest priority, and at the scene the patient was unconscious and needed care with highest priority. Exclusion criteria of calls were; over- triage calls due to the safety margin expected in the EMS. And calls caused by events such as traffic accidents, house fires or violence were excluded since these calls are assessed according to the event and not by the caller’s symptom description [19]. Calls regarding intra-hospital transport were also not included for analysis since these calls are usually carried out on behalf of physicians at hospitals. An assumption was that this purposeful sample would give a variety of calls to analyse. The sampling of calls started on the 1st of March 2011 and ended on the 31st of March 2011. The number of 50 calls in each group was considered to be sufficient and manageable for a qualitative analysis intended to identify overall factors influencing the RN assessment of calls to the EMCC [18]. A description of the sampled calls is given in Table 1.

**Table 1:** **Included calls for analysis**

**Group 1; Agreement between EMCC nurse and ambulance nurse (n = 50)**		**Group 2; Disagreement between EMCC nurse and ambulance nurse (n = 50)**		
**Dispatched as;**	**n**	**Dispatched as;**		**Feedback from ambulance nurse**
Stroke	18	Undefined problems	23	Fever/infection (8)
Chest pain	6			Breathing difficulties (8)
Convulsions	5			Stroke (2)
Breathing difficulties	4			Cardiac arrest (1)
Bleeding – no trauma	3			Unconscious (1)
Poisoning	3			Ear/nose/throat problems (1)
Undefined problems	3			Abdomen/urinary problems (1)
Child illness	2			Chest pain (1)
Diseases or injuries o limbs	2	Chest pain	7	Undefined problems (3)
Allergy	1			Breathing difficulties (3)
Birth/pregnancy	1			Fever/infection (1)
Diabetes	1	Breathing difficulties	6	Chest pain (3)
Headache/dizziness	1			Undefined problems (2)
				Unconscious (1)
		Abdomen/urinary	5	Chest pain (2)
		problems		Fever/infection (2)
				Undefined problems (1)
		Fever	3	Breathing difficulties (2)
				Undefined problems (1)
		Bleeding no trauma	2	Abdomen/urinary problems (1)
				Undefined problems (1)
		Back problems	1	Breathing difficulties (1)
		Diseases or injuries to		
		limbs	1	Accident (1)
		Psychiatric	1	Dead at scene (1)
		Headache/dizziness	1	Stroke (1)

### Analysis

Qualitative content analysis described by Elo and Kyngäs was used to identify factors involved in assessing an emergency call [20]. The two data groups were separately analysed but in the same way. The first part of the analysis consisted of listening to and transcribing the tape recordings. All identified emotions and sounds in the background (i.e. crying, breathing sounds) were noted in the text. To evaluate the emotion and cooperation of the caller, an emotion, content and cooperation score described by Clawson and Sinclair was used to classify the caller’s emotion during the call, as follows: (1) normal conversational speech (2) anxious but cooperative (3) moderately upset but cooperative (4) uncooperative not listening, yelling and (5) uncontrollable, hysterical [21]. The classification of the caller’s emotion was conducted while listening to the tape-recordings. The second part of the analysis, which aimed to describe the calls, contained a recording of nominal data: duration of the call, care seeker’s age, language difficulties, who made the call, relation to the patient, adherence to protocol, and information received about what the patient was doing during the call. The adherence to the protocol of the Swedish Medical Index [13] was defined as “yes”, if any information about the patient’s breathing and consciousness were identified during the call, and “no”, if nothing during the call could be identified as information to the RN about the patient’s breathing and consciousness. All the nominal data were assessed during the transcriptions of the calls and when reading the transcribed text. Then, all the transcribed material was read several times to obtain a sense of the whole. Thereafter, an open coding of the transcribed calls was conducted: underlining was used to emphasize the text that described the different aspects of assessing the call to the EMCC. Headings were written down to describe the content of the underlined sections and a note was made in the headings if the content was related to the RN or the caller. After this open coding, a coding sheet with all headings was created and then categorized into broader and higher-order categories (sub- categories). The sub- categories with similar content related to the RN or the caller were then grouped together as generic- categories [20]. The identified generic- categories in each group were then compared in an attempt to identify differences in the call where the RN did or did not identify the callers’ need of care. The differences became the main categories. The final step in the analysis consisted of verifying that the results were representative of the collected calls. This was done by going back and reading the transcribed calls.

## Results

The analysis identified four main categories as overall factors influencing the assessment of the call to the EMCC: “Barriers” and “Opportunities” related to the caller or the RN.

**Figure 1:**
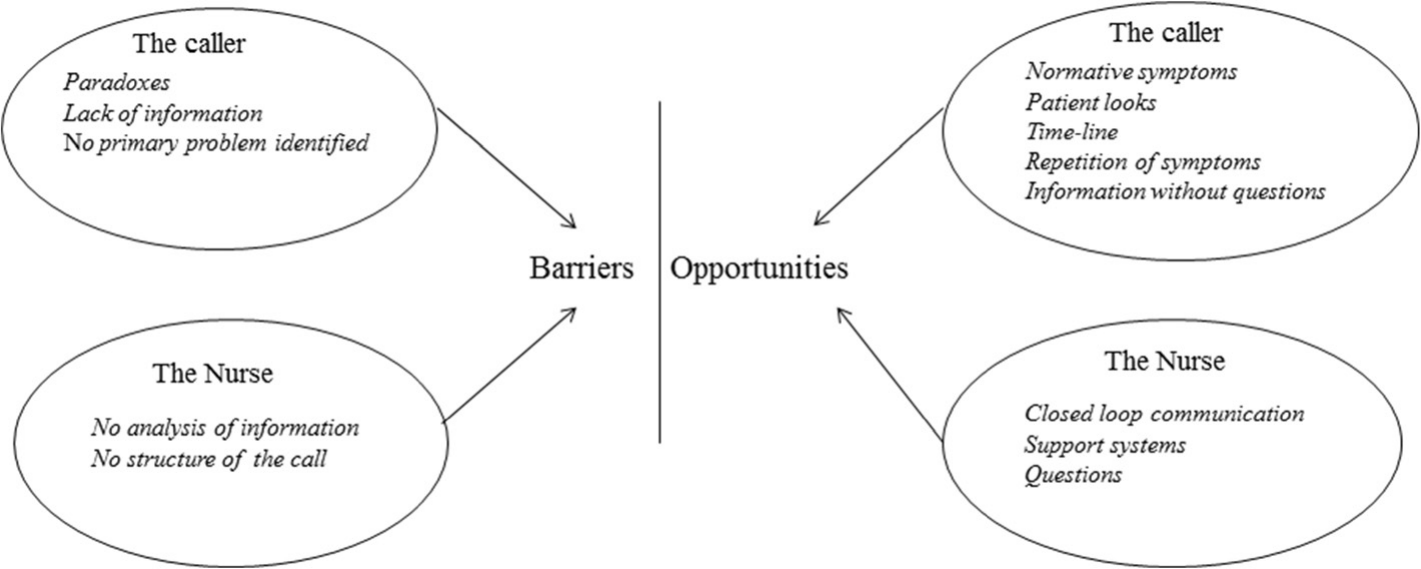
**Identified barriers and opportunities of assessing calls.**

There were three identified generic- categories of barriers related to the caller, and five categories of opportunities. There were two identified generic- categories of barriers in assessing the calls related to the RN, and three categories of opportunities, as shown in Figure 1.

An overall description of the analyzed calls as displayed in Table 2.

**Table 2:** **Description of the calls**

	**Same assessment of the patient’s primary problem and priority (n = 50)**	**Mismatch in the assessment concerning priority and patient’s primary problem (n = 50)**
**Emotion and content score between 1-5**	**Mean**	
	1,28	1,14
**Call to;**	**n (%)**	
Emergency number	43 (86)	33 (66)
Elective ambulance transport	7(14)	17 (34)
Bystanders	9 (18)	5 (10)
**Who made the call;**	**n (%)**	
Health care providers*	19 (38)	21 (42)
Relatives	15 (30)	16 (32)
Patients	7 (14)	8 (16)
**Duration of the call**	**Min:sec**	
Range	01:07-10:35	01:08-07:16
Median	02:36	03:26
Mean	03:16	03:42
**First symptom description**	**Seconds**	
Range	5-83	5-146
Median	15	30
Mean	22	36
**Patient’s age**	**Years**	
Range	0,2-103	15-93
Median	72	84
Mean	66	72
Not known	(n)16	(n) 11
**Previous medical history mention during call**	**n (%)**	
Yes	28 (56)	39 (78)
No	22 (44)	11 (22)
**Sound of patient breathing**	**n (%)**	
Yes	21 (42)	11 (22)
No	29 (58)	39 (78)
**What patient is doing**	**n (%)**	
Yes	32 (64)	21 (42)
No	18 (36)	29 (58)
**Adherence to assessment protocol**	**n (%)**	
Yes	38 (76)	29 (58)
No	12 (24)	21 (42)
**Language difficulties**	**n (%)**	
Yes	11(22)	5 (10)

*Health care providers: Primary health care nurses, home care personnel, personal assistants and assistant nurses.

### Opportunities related to the caller

#### Normative symptoms

An identified opportunity in assessing the call to the EMCC was the normative symptom description by the caller, when the callers’ symptom descriptions were presented immediately in the call and exactly as the literature and assessment protocol describes specific medical conditions. *“…for 30 minutes my wife has been unable to move the right side of her body.*

*I had to carry her to our bed, she cannot walk”.* This example shows how the caller’s description of the patient’s ongoing symptoms is exactly as in the literature and the assessment protocol describing a patient with a suspected stroke.

#### Patient’s look

An opportunity in assessing the call appeared when the caller describes how the patient actually looks and how the skin is feeling, e.g. *“His facial color is actually gray and the skin is cold but sweaty…”* The caller description was colorful and gave an impression of how the patient actually looked, and the grayness and clamminess could indicate a critically ill patient according to the medical literature.

#### Time-line

Besides a clear description of the patient’s symptoms and appearance, a time-line description was identified as an opportunity in assessing the call. *“I saw him an hour ago and he was ok… now he has pain in his chest, is wheezing and wants to vomit”* (home care personnel)*.* Here, the caller describes how the patient became ill within the timeframe of 60 minutes, and the symptoms may indicate a severe illness. Another way of describing the time line was when the caller described the patient’s previous condition and how the patient was at the moment *“…normally he can talk and walk, now he cannot stand by himself …”* if the caller gave the information in combination with a time-line it became an opportunity to identify the patient’s need of care.

#### Repetition of symptoms

When the callers repeated the symptoms several times and they also sometimes changed the description of the same symptom, this was identified as an opportunity. *“…she cannot speak normally… something is wrong with her head… she does not understand… she does not follow my instructions; she just looks at me as if I’m the confused one when I’m talking ….”* This example shows how the callers repeated the patients’ symptoms’ several times but in different ways.

#### Information without questions

When the caller gave more information than requested concerning the patient’s previous medical history and this information confirmed the given symptom description it was an opportunity in assessing the call. An example: a wife calls and says that her husband is lying on the floor and he is unconscious, the RN ascertains that the patient is breathing and without any questions the wife says that the husband has been having problems taking his medicine for epilepsy for some days. And when the overheard sounds and the patient talking during the call confirm the information and given symptom description then it also support the description of the on-going symptoms.

### Opportunities related to the registered nurse

#### Closed loop communication

An identified opportunity in assessing the call to the EMCC was when the RN used closed loop communication, meaning the nurse repeated and/or concluded the information given by the caller in some form. The caller confirmed the conclusions the RN made from the given information and if the RN made a conclusion that was not in line with the caller’s information the caller corrected the RN.

#### Support systems

In the calls where the RN used some form of support for assessing the call, such as contacting an expert in the area (i.e. midwife, poisoning emergency line) or used specific assessment instruments (i.e. the Face Arm Speech Test) this was also an identified opportunity of assessing the call.

#### Questions

The communication strategy of using questions related to the information given by the caller was another opportunity. *“Caller:… there are a lot of bubbles in his mouth… RN: Can he talk to you?* When no answers were given by the caller, the RN repeated or re-formulated the question until answers were given, an example. *“Caller: he is just lying down … RN: can he talk … ? RN: can you see if he’s breathing …? RN:… can you see if his chest is moving …? Caller:…he is looking at me … RN: yes, but is he breathing …? Caller: yes… yes… now I see he is breathing and he is trying to sit up…”*

### Barriers related to the caller

#### Paradoxes

Through the analysis of the calls, different types of paradoxes related to the caller were identified as barriers. The caller’s information was contradictory concerning symptom descriptions and the background sounds in the calls. For example, the patient could say

“*I cannot breathe”* but the sound of breathing was normal and there was no sign of dyspnea during the call. Another paradox seemed to arise when the patient was relatively young and the symptom descriptions indicated severe illness, but the caller was not anxious. The third paradox arose when the caller made the call about one problem and then described symptoms of something else. An example: the caller described a problem of nose bleeding for two hours (taking anticoagulantia). The paradox appeared when the patient then described a pressure over the chest and pain radiating to the left hand, but this was not the main problem according to the caller.

#### Lack of information

Another identified barrier in assessing the call was when there appeared to be some problem concerning gathering information for an assessment, resulting in a lack of information.

The reasons were: the caller could not see the patient, the caller did not answer the RN’s questions, or the amount of information given by the caller was unmanageable and the patient’s primary problem was not identified from what was said. Lack of information was also caused by the patient being unable to explain the problem because of previous cerebral injury.

#### No primary problem

Several of the calls were assessed by the nurse at the EMCC as ‘undefined problems’. In these calls the callers did not describe a single problem or a time line; instead they described several symptoms, for example. *“… my mum looks so thin, so tired… she has a severe cough and is breathing heavily… she is dizzy and woozy… she has cancer”.* The caller described a sick patient but most of the symptom descriptions were vague, and no single acute problem was presented. The same barrier, no primary problem, was identified in the calls where the patient was older than 80, the caller described a history of multiple illnesses, the patient had recently become confused, the patient had a fever and had vomited, or had recently become unable to walk or stand as the caller describes a sick problem but no single acute problem was presented.

### Barriers related to the registered nurse

#### No analysis of information

An identified barrier related to the RN appeared when the RN seemed to focus on the primary problem presented by the caller instead of focusing on and analyzing the more severe symptom description (or breathing sounds) presented in the call. For example: the call to the EMCC was concerning a urinary problem, but during the call, the patient’s breathing sounds were clearly obstructive. The RN focused on the patient description of the urinary problem, and no questions were asked about whether the patient was experiencing any respiratory problems. In six of the calls the information concerning the patient’s breathing, circulation and consciousness was collected by the RN, *“… a respiratory rate of more than 50 … she is bleeding from the nose… the patient doesn’t look good, she is gray …”* (a call made from a nursing home). The RN repeated the information and asked questions about the given information but did not take any action based on the collected information.

#### No structure to the call

Another identified barrier was when there was no structure to the call, for example the RN asked more than one question in the same sentence, and the caller answered just one of the questions, and no follow-up questions were asked. Another barrier was when the RN did not take command in the call and the caller lost focus on the primary problem and started to talk about other things.

In some of the calls there was no structure to assist in clarifying the situation regarding the patient’s breathing, circulation and/or consciousness. Instead, these calls focused on counseling, the patient's previous medical history, drugs, whether there was actually a need for an ambulance, and social factors that may have contributed to the patient’s illness.

This became a barrier in assessing the call. No structure to calls also appeared in calls made by other professional health care givers, when they started the call with “… *the doctor has decided… this is just a transportation of a patient to the hospital…”* Someone else had decided that the patient should be transported by ambulance and the EMCC nurse did not make any structured assessment of patient status.

## Discussion

This study describes both barriers and opportunities related to assessing the call to the EMCC. The main discrepancies appeared in communication strategies used by the RN and symptom descriptions made by the caller. The communication strategies used by the RN could become an opportunity in the best case, but also a barrier in the worst case. Nevertheless, when the barriers are related to the RN there is a possibility for improvement work. It is harder, but maybe not possible, to influence the callers’ ways of expressing themselves.

When assessing the emergency call, an opportunity seems to be use of a closed loop communication strategy by the RN, with conclusions and questions related to the information given by the caller. The use of follow-up questions and conclusions may be a way of making sense of and sorting out the information given by the caller. However, since closed loop communication is described as supporting precise and accurate communication [22] it may serve as a tool to improve patient safety in the EMCC. Whether the use of closed loop communication is a specific skill developed in an ad hoc way based on the RNs’ professional background and experience in managing interactions with callers as argued by Pettinari & Jessop [23] is not determined by this study but a question arises; is it possible to develop assessment protocols supporting problematic communication situations?

Knowing the patient’s previous medical history may be an opportunity or a barrier when assessing the call. The information may support the caller’s symptom description and allow the RN to identify the patient’s need of care. It could also be that the previous medical history may be a barrier. If the RN does not select the information given by the caller in a proper way, the information may disappear in the information flow. The use of closed loop communication strategies with conclusions may be a possible way to extract the information under such circumstances. However, as our results indicate, assessing and triaging the patient’s illness by means of a phone call is a complex task.

Salk et al. describe poor agreement between assessments of the same patient in person and over the telephone. In their study, the accuracy of telephone assessment was not enhanced by the use of chief complaint-based protocols or by being informed about the patient’s vital signs [24]. This result could be congruent with our results *“… a respiratory rate of more than 50”*, it may not be a lack of the measured vital signs; other factors influence the assessment. Another barrier in assessing a call seems to be when the RN at the EMCC does not clarify the situation regarding the patient’s breathing and/or consciousness; instead the RN focuses on other matters. This result is congruent with the study of Bång et al. where the EMD asked only 41 percent of the callers if the patient was breathing normally [11]. Previous studies describe protocol compliance as an important factor for successfully triaging in the EMCC [3,6]. In our study, there were some cases where although the RN followed the assessment protocol and ascertained whether the patient was breathing and/or conscious, nevertheless, the RN did not treat the call as a high priority case. There may be more than just protocol compliance involved in the assessment of the patient over the phone. Our study does not reveal factors outside the call, but factors such as the availability of ambulances, or calls waiting to be answered may also interfere in the decision-making. It could also be that the first call taker misled the RN in their assessment. Further research is needed to identify and explore these factors.

Another opportunity for assessing the emergency call appeared when the symptom descriptions were given by the caller as the literature describes. When the symptom descriptions were vaguer, and were not as the assessment protocol describes, this became a barrier. Whether this means that the assessment protocol needs to be developed or whether there are communication strategies that need to be developed is unclear from this study result but there is a need to develop support tools for the RN assessing the calls. Otherwise, the RNs cannot improve their assessments. Different paradoxes were identified as barriers during the calls - paradoxes that have not been described in previous studies. This result may indicate that there are additional aspects of the calls to the EMCC to be considered. The knowledge of how the caller may express paradoxes during the call can be useful when educating RNs at the EMCC, but these identified paradoxes also need to be examined in future studies. Barriers are commonly described as language and communication problems [8,25,26]. In this study, language problems were not identified as a barrier, although a third of the calls were made by a non- native speaker. Another barrier to assessing the call arose if the patient was elderly (older than 80). This result may indicate an issue with geriatric training for the RN at the EMCC or a need to describe the geriatric patients more clearly in the Swedish Medical Index. A previous study has found that increased age and a mental change from an assumed baseline are important clinical factors to consider when identifying a patient’s need of care [27], and this is not clearly stated in the Swedish medical index [13]. However, the signs and symptoms available to the RN for assessing the call are reduced to those the caller describes. Maybe we need to increase our understanding of how ill patients communicate with nurses, especially how this is done by phone. The questions to the caller may not always be as clear as we think. Further studies are needed to clarify this.

The results should be interpreted in the context of the following methodological limitations. The purposeful case sampling was intended to provide an insight into factors that influence the assessment, rather than to determine if the assessment made by the RN at the EMCC was right or wrong. The purposeful sample included a variety of calls both from lay persons and calls from various health care providers. Calls to the emergency number and calls for elective ambulance transport were both included for analysis. This can be seen as a limitation since the analysed calls were not a heterogeneous group. Another limitation of this sampling is that the calls for elective ambulance transport or calls to the emergency number may have been assessed differently by the RN at the EMCC. The RN may have different expectations concerning the severity of the call to the different phone numbers. On the other hand, the aim of the purposeful sampling was to achieve a variety of calls (as in reality) to analyse, and all healthcare-related calls to the EMCC should be evaluated by the RNs regardless of phone number used and who makes the calls. However, in order to understand the assessment differences in assessing calls to different phone number at the EMCC, further research is required. Nevertheless, the result shows that there are barriers and opportunities in the calls from both laypersons and health care providers, and further research is needed to explore the reasons. Not including the over triage calls when we conducted the purposeful sampling may have caused a lack of valuable information in the result. The decision not to include these calls was due to the safety margins that should be in EMS systems, and the over triage calls were expected to contain previously described difficulties in assessing certain calls to the EMCC. However, we do not know if this assumption was true. We listened to and transcribed the recorded calls, and in this study it was a limitation that there was no opportunity to ask the caller and RN clarifying questions when clarification was needed to explain what had happened during the call. By using a talk aloud method [18], listening to the recorded tapes and encouraging the RN to reason about the assessment could have clarified some of the paradoxes. Analysing calls with qualitative content analysis yields an in-depth understanding rather than an empirical generalization of the results [18]. During the analysis, peer debriefing and member checks were conducted among the authors. To achieve content validity [28] the identified barriers and opportunities were critically discussed with an experienced RN at the EMCC. The identified generic and main categories were also discussed with approximately 50 of the RNs from different EMCCs around Sweden. The discussions were conducted in smaller groups when the results were presented during the RNs’ annual training arranged by their employer, SOS Alarm. The discussions with RNs at the EMCC confirmed the results, and the nurses agreed with the opportunities and barriers identified in this study.

## Conclusion

Barriers in assessing the call to the EMCC were associated with contradictory information, absence of a primary problem, or the structure of the call. Opportunities were associated with a clear symptom description that was also repeated, and with the RN’s use of different communication strategies such as closed loop communication.
